# Gaps in dementia knowledge: a nationwide study of public awareness and misconceptions in Bulgaria

**DOI:** 10.1111/psyg.70016

**Published:** 2025-02-27

**Authors:** Sophia Lazarova, Dessislava Petrova‐Antonova

**Affiliations:** ^1^ GATE Institute Sofia University “St. Kliment Ohridski” Sofia Bulgaria; ^2^ Faculty of Mathematics and Informatics Sofia University “St. Kliment Ohridski” Sofia Bulgaria

**Keywords:** dementia, health literacy, health promotion, public knowledge, risk reduction

## Abstract

**Background:**

Dementia is a global health concern which can be mitigated by primary prevention and improved literacy. Effective educational initiatives are informed by studies of dementia knowledge. However, most of these studies are conducted in high‐income countries, leaving the Balkan region underrepresented. This study aimed to conduct the first investigation of dementia knowledge among the Bulgarian population, exploring recognition of symptoms, general dementia knowledge, and risk factors awareness.

**Methods:**

Using an online survey we assessed the following components of knowledge: (i) recognition of dementia symptoms from a vignette; (ii) dementia literacy measured with the Dementia Knowledge Assessment Scale (DKAS); and (iii) knowledge about dementia risk factors. Demographic characteristics, previous experience with dementia, and patterns of informing about dementia were also considered in the study.

**Results:**

One thousand, eight hundred and ninety‐six adults (mean age = 44.99; 51.79% female) completed the survey. Half of the respondents correctly recognised dementia symptoms from a vignette. The average DKAS score was 9.51. Dementia knowledge was linked to education, marital status, employment, ethnicity, experience with dementia, and informational sources. 56.7% of the respondents thought dementia was a normal part of ageing and 74.8% did not know a healthy lifestyle reduces the risk of dementia. The average number of identified risk factors was eight (out of 17), with many mistakenly citing dental fillings, laziness, and witchcraft as contributors to dementia.

**Conclusions:**

The Bulgarian society has a poor understanding of dementia, highlighting the need for improved awareness and education. Policy‐makers should prioritise dementia as a social issue and take coordinated actions to educate society and eradicate harmful misconceptions.

## INTRODUCTION

Dementia is a fatal disease, causing a gradual decline in cognitive function, impaired social and occupational abilities, and loss of independence.^1^ The number of people living with dementia worldwide in 2019 was estimated at 57 million and is projected to reach 153 million by 2050.^2^ Following the broader European and world trends, cases of dementia in Bulgaria are expected to rise over the next decades.^3^ Considering the rapidly ageing population of the country, fourth in the world for its rate of ageing,^4^ an increasing proportion of the Bulgarian population will be at higher risk of developing dementia. Meanwhile, the European Dementia Monitor report from 2023 ranked Bulgaria's response to dementia next‐to‐last among 37 European countries.^5^ Given these figures, immediate actions are needed to contain the impact of dementia, as the increasing number of cases may easily overwhelm the underprepared social infrastructure of Bulgaria.

Dementia literacy, defined as knowledge and beliefs regarding dementia that aid recognition, management, or prevention, is central to improving care and lowering incidence.^6^ A systematic review from 2019 identified a correlation between health literacy and the post‐mortem accumulation of plaques and tangles in patients with Alzheimer's disease, suggesting that lower health literacy may be associated with an increased risk of developing dementia.^7^ Several other studies reported a connection between lower health literacy and higher dementia prevalence and incidence.^8^, ^9^, ^10^ The importance of dementia literacy is further highlighted by the fact that knowledge levels were shown to influence attitudes and behaviours toward sick individuals,^11^, ^12^ quality of care,^13^, ^14^, ^15^ stigmatisation,^16^, ^17^ and willingness to seek help.^18^, ^19^ Therefore, adequate public knowledge may improve disease management and primary prevention, ultimately contributing to a better social environment for those affected by the disease.

The existing literature on dementia knowledge consistently reports low‐to‐moderate levels of knowledge paired with the commonly occurring belief that dementia is a normal part of ageing.^20^, ^21^, ^22^ Additionally, previous studies reported major gaps in knowledge about modifiable risk factors and disease prevention^21^, ^23^, ^24^, ^25^, ^26^ with cardiovascular factors such as dyslipidaemia, hypertension, heart disease, obesity, alcohol, healthy diet, diabetes, and so forth, being least understood.^21^, ^23^, ^25^, ^26^, ^27^ However, most of these studies were conducted in high‐income countries, leaving a gap in the literature regarding dementia knowledge in middle‐ and low‐income regions.^21^

To the best of our knowledge, dementia literacy has never been investigated in Bulgaria. Furthermore, apart from two small studies in Croatia^28^ and Greece,^29^ the state of dementia literacy in the Balkans has been largely unexplored. We aim to fill this gap in the literature by investigating lay knowledge about dementia among the Bulgarian public. Given that dementia is not considered a health priority by the Bulgarian authorities, and the lack of a national dementia plan and coordinated outreach efforts, we anticipate poor levels of dementia literacy among the general population. However, the significance of this research is not in confirming the low levels of dementia literacy, but in identifying specific misconceptions and gaps in knowledge about the disease. Thus, in addition to utilising the commonly used Dementia Knowledge Assessment Scale (DKAS),^30^ the present study investigates two socially significant aspects of dementia literacy–recognition of dementia symptoms and knowledge about dementia risk factors. The ability to recognise the manifestations of the disease is critical in terms of receiving a timely diagnosis and appropriate treatment. Similarly, having a sound knowledge of dementia risk factors is a prerequisite for adequate personal prevention of the disease. Therefore, identifying existing knowledge gaps in these two domains would serve clinicians, policy‐makers, educational initiatives, and governmental authorities in their efforts to educate society and improve awareness. To further inform such efforts, we aim to identify groups within Bulgarian society that might be prone to false beliefs and low dementia literacy levels. Thus, among other sociodemographic features, we are set to investigate the role of having previous experience with dementia and various information acquisition practices on literacy levels.

## MATERIALS AND METHODS

### Procedure

Dementia knowledge was assessed as a part of a larger study, investigating the lifestyle, and daily habits of Bulgarians. An online survey, implemented with the Computer‐Assisted Web Interview (CAWI), was conducted between 23 February and 28 March 2024. To prevent priming effects, the title of the survey did not contain any references to, or mentions of, dementia. The technical development of the online survey and the recruitment of participants was outsourced to an agency for social surveys and public opinion polls. All communication with the respondents was handled by the agency. Questionnaire completion was possible only once from a single IP address. Respondents were provided with an informational sheet and were required to provide their consent to continue to the survey. Only fully completed survey forms were recorded. The survey was available only in Bulgarian.

### Participants

The selection of participants was based on quota sampling in order to obtain a nationally representative sample of Bulgaria's adult population. The respondents were sourced from the database of the contracted agency and were contacted via email. A total of 2017 participants completed the survey. All participants were Bulgarian citizens, currently residing in Bulgaria.

### Measures

The questionnaire included the following components: recognition of dementia symptoms from a vignette, assessment of dementia knowledge and risk factors knowledge assessment. To account for possible influences, several questions regarding previous experience with dementia and sources used to learn about dementia were also included.

#### Demographic characteristics

The following demographic data were collected: age, gender, ethnicity, education, place of residence, marital and employment status.

#### Previous experience with dementia

Three questions pertaining to previous experience with the disease were included in the questionnaire. Each of them was assessing one of the following aspects: experience as a caretaker, having a relative who is/was sick from dementia, and having a profession that requires contact with dementia patients.

#### Informational sources

The quality and variety of informational sources used by each participant were evaluated with the following multiple‐choice question: ‘Which of the listed sources do you use to inform yourself about dementia?’ Eight possible options were provided with an auxiliary option ‘Other’. The list of options included the following items: formal sources such as scientific literature, lectures, and medical specialists; conventional media sources such as television and radio; social media and internet searches; social sphere sources such as friends, family, as well as direct contact with sick individuals.

#### Recognition of dementia symptoms

Recognition of dementia symptoms was assessed with a vignette describing a person (female protagonist named Maria) with moderate symptoms of Alzheimer's disease. The content of the presented vignette was as follows:

‘*Maria is a 75‐year‐old widow*. *Her family thinks that her memory is getting progressively worse*. *She tends to tell the same stories over and over, and frequently reminisces about her husband*, *talking as if he were still alive*. *She appears to forget what she has been told within minutes*. *She follows conversations poorly and occasionally gets confused*, *angry*, *and aggressive*. *Her family has taken over her banking because she was not paying her bills and could no longer balance her cheque book*. *They also have hired a cleaner because her home was getting very dirty*. *They worry that she has not been showering regularly*. *She stopped seeing her friends over the last 5 years and very rarely leaves her home now*. *She told her family that a strange man had broken into her house and is still living in the spare room*, *but they could not find any evidence for this*’.

The vignette was sourced from a work of Low and Anstey, published in 2009.^31^ The same vignette was reused in a later study by the same group^32^ as well as in several other studies that adapted the approach.^33^, ^34^ The original vignette was translated into Bulgarian without content alterations except for modifying the name of the protagonist from ‘Mary’ to ‘Maria’, a common name in Bulgaria. Participants were presented with the vignette and then asked the following open‐ended question: ‘What would you say is wrong with Maria (if anything)?’ This question was followed by an auxiliary question – ‘Do you believe that Maria has some mental illness?’ (yes/no answer). To avoid priming effects, neither the word ‘dementia’ nor any related terms were present in the questionnaire prior to the recognition task.

#### DKAS

A structured measure of dementia knowledge was obtained with the DKAS.^30^, ^35^ The DKAS is a 25‐item scale, containing correct and incorrect statements about dementia. Participants were asked to express their (dis)agreement with each of the statements using a five‐point Likert scale that ranged from ‘false’ to ‘true’ with an auxiliary option ‘I don't know’. The DKAS consists of four subscales: ‘Causes and characteristics’, ‘Communication and behaviour’, ‘Care considerations’, ‘Risks and health promotion’. The questionnaire has a total score of 50 points and is scored by the following system: two points for an answer ‘true’ to a truthful statement or an answer ‘false’ to a false statement; one point for an answer ‘probably true’ to a truthful statement or answer ‘probably false’ to a false statement; zero points for an answer ‘true’ or ‘probably true’ to a false statement; zero points for an answer ‘false’ or ‘probably false’ to a true statement; and zero points for an answer ‘I don't know’. The original questionnaire was adapted in Bulgarian and then internally evaluated by domain experts for wording and accuracy of terminological translation.

#### Recognition of dementia risk factors

Each participant was presented with a list of 30 items and asked to rate the contribution of each item to the development of dementia. Participants rated the items on a five‐point scale ranging from ‘Contributes’ to ‘Does not contribute’ with an auxiliary option ‘I don't know’. Each of the items was either an empirically supported risk factor (true risk factor), a plausible risk factor with emerging evidence for its connection to the development of dementia, or a sham factor with no scientific evidence relating it to the development of dementia.

The list included the two non‐modifiable risk factors for dementia (old age and genetic predisposition) and 15 modifiable risk factors, most of which were based on the Lancet Commission report (obesity, diabetes, hypertension, lack of physical activity, fewer years of education, smoking, depression, lack of social contact, hearing loss, high alcohol consumption, head trauma).^36^ This list was enriched with five additional factors, commonly accepted as modifiable risk factors for dementia, namely, cardiovascular disease,^37^, ^38^, ^39^ stroke,^40^, ^41^ unhealthy diet,^42^, ^43^, ^44^, and lack of cognitive engagement.^45^, ^46^, ^47^

To account for the plausible effect of infections on the risk of dementia, the following three items were included as factors supported by partial evidence: bacterial infection, viral infection, and parasitic infection. Several viral infections have been previously implicated as plausible contributors to dementia and Alzheimer's disease, including viral influenza, viral pneumonia, herpes simplex encephalitis, hepatitis C virus, genital warts, and the Epstein–Barr virus.^48^, ^49^, ^50^, ^51^ Similarly, common bacterial pathogens have been associated with an increased risk of dementia^52^, ^53^ and recently emerging evidence elucidated the role of the single‐celled parasite *Toxoplasma gondii* as a potential contributor to the genesis of Alzheimer's disease.^54^, ^55^

Finally, 10 items without empirical evidence were included to capture common cultural beliefs (amalgam dental fillings, laziness, stress, aluminium, curse/witchcraft, aspartame, fate, vaccines, poverty, and weakness of character). Some of the items were sourced from the Alzheimer's Association website^56^ and informally consulted with physicians; others were sourced from existing literature and the local culture.^33^, ^57^

A quantified measure of knowledge about dementia risk factors was obtained as a sum of the successfully identified empirically supported risk factors. A successful identification was considered answering ‘Contributes’ and ‘Probably contributes’ to an empirically supported risk factor. Thus, the maximum score was 17 points.

### Analysis

Data processing and analysis were conducted using R Studio (v2023.03.1 Build 446). The default level of statistical significance was 0.05. Proportions, means and standard deviations (SDs) were used as descriptive statistics of the sociodemographic variables, symptoms recognition (vignette task), and dementia knowledge estimates. The normality of dementia knowledge data was checked by assessing skewness and kurtosis, which were in the acceptable ranges. Since our sample is larger than 300 observations, the normality of data was determined by the absolute values of skewness (≤2) and kurtosis (≤4).^58^ Pearson's *ꭓ* ^2^ tests of independence were performed to investigate the associations between sociodemographic factors and the ability to recognise dementia symptoms from a vignette. Significant results were further examined with a *post hoc* analysis of residuals. One‐way analysis of variance tests were used to investigate the differences in dementia knowledge and knowledge about risk factors between groups defined by sociodemographic characteristics and the number of used informational sources. *Post hoc* analyses were performed with Tukey's Honestly Significant Difference (HSD) test to further unveil the differences between the groups. Two‐sided Welch's *t*‐tests were performed to investigate the effects of having previous experience with dementia and using high‐quality sources of information on dementia knowledge and knowledge about risk factors. The association between general dementia knowledge and the capacity to recognise dementia symptoms from a vignette was assessed with a two‐sided *t*‐test. Finally, one‐tailed binomial tests were used to test whether the proportions of correctly identified true risk factors and sham items were higher than chance.

### Ethics approval

The study was performed according to the ethical standards outlined in the Declaration of Helsinki. All participants voluntarily enrolled after being presented with an informational sheet and explicitly providing their consent for participation. Access to the questionnaire was granted only to participants who provided their consent. The general data protection regulation (GDPR) in a research context^59^ was respected through the confidentiality and anonymity of the data. Ethics approval was obtained from the Ethics Committee of the Sofia University ‘St. Kliment Ohridski’ (no. 70‐123‐96/19.01.2024).

## RESULTS

### Characteristics of study participants

A total of 2017 respondents completed the survey. One hundred and twenty‐two of them were excluded due to a consistent provision of neutral answers, thus our analytical sample included 1896 participants (*N* = 1896; mean = 44.99 years; SD = 12.74 years, 51.79% female). A little more than 17.00% of the participants had previous experience with taking care of someone with dementia, 28.80% reported having a close relative affected by dementia and 3.64% reported having a profession that requires personal contact with dementia patients (Table 1).

**Table 1:** Summary of sample characteristics (*N* = 1896). Maximum possible Dementia Knowledge Assessment Scale (DKAS) score‐50

Gender	*n*	Total (%)	Vignette correct (*n*)	Mean DKAS score	Mean risk factors
Male	914	48.21	469	9.44	7.40
Female	982	51.79	479	9.56	7.60
Age					
18–35 years	577	30.43	285	9.40	7.88
36–54 years	756	39.97	378	9.58	7.43
55+ years	563	29.70	285	9.53	7.22
Region of residency					
Southwest region	550	29.00	277	9.48	7.56
Southeast region	284	14.98	145	9.03	7.29
Northeast region	243	12.82	106	9.58	7.45
Southcentral region	374	19.73	191	9.72	7.63
Northcentral region	243	12.82	122	10.07	7.54
Northwest region	202	10.60	107	9.12	7.44
Type of living area					
Urban	433	22.8	207	9.33	7.40
Rural	251	13.2	118	9.26	7.27
City‐Administrative centre	1212	63.9	623	9.63	7.59
Education					
Primary	2	0.1	1	6.50	2.50
Secondary	823	43.4	383	8.60	6.74
Bachelor's degree	528	30.7	295	10.23	8.04
Master's degree	487	25.7	268	10.20	8.18
PhD	2	0.1	1	10.50	6.50
Employment					
Full‐time	1254	66.14	643	9.72	7.73
Part‐time	219	11.55	102	10.17	8.46
Unemployed	70	3.69	32	5.59	5.17
Retired	104	5.49	53	8.54	5.46
Other	249	13.13	118	9.40	7.04
Marital status					
Single	350	18.46	171	8.55	6.67
In relationship	599	31.59	309	9.24	7.48
Married	684	36.08	328	10.27	8.13
Divorced	202	10.65	105	9.06	6.82
Widowed	61	3.22	35	10.66	7.85
Ethnicity					
Bulgarian	1624	85.65	816	9.24	7.25
Turkish	216	11.39	98	10.80	8.66
Roma	16	0.84	8	13.19	11.44
Other	38	2.00	25	12.42	10.11
Will not answer	2	0.11	1	7.00	8.00
Personal experience with dementia					
Cared for someone with dementia	Y: 328 N: 1568	Y: 17.30 N: 82.70	Y: 160 N: 788	Y: 10.90 N: 9.22	Y: 7.42 N: 7.52
Have a diagnosed relative	Y: 546 N: 1350	Y: 28.80 N: 71.20	Y: 274 N: 657	Y: 10.78 N: 9.00	Y: 7.53 N: 7.49
Profession requires contact	Y: 69 N: 1827	Y: 3.64 N: 96.40	Y: 45 N: 903	Y: 12.46 N: 9.40	Y: 8.96 N: 7.45
Quality of informational sources					
Using formal informational sources	Y: 378 N: 1518	Y: 19.94 N: 80.06	Y: 187 N: 761	Y: 11.28 N: 9.07	Y: 8.16 N: 7.34
Quantity of informational sources					
Using less than 3 sources	1221	64.40	581	8.72	7.29
Using between 3 and 5 sources	656	34.60	356	10.83	9.93
Using more than 6 sources	19	1.00	11	14.47	6.42

Maximum possible number of correctly identified risk factors – 17.

### Sourcing information about dementia

**Figure 1:**
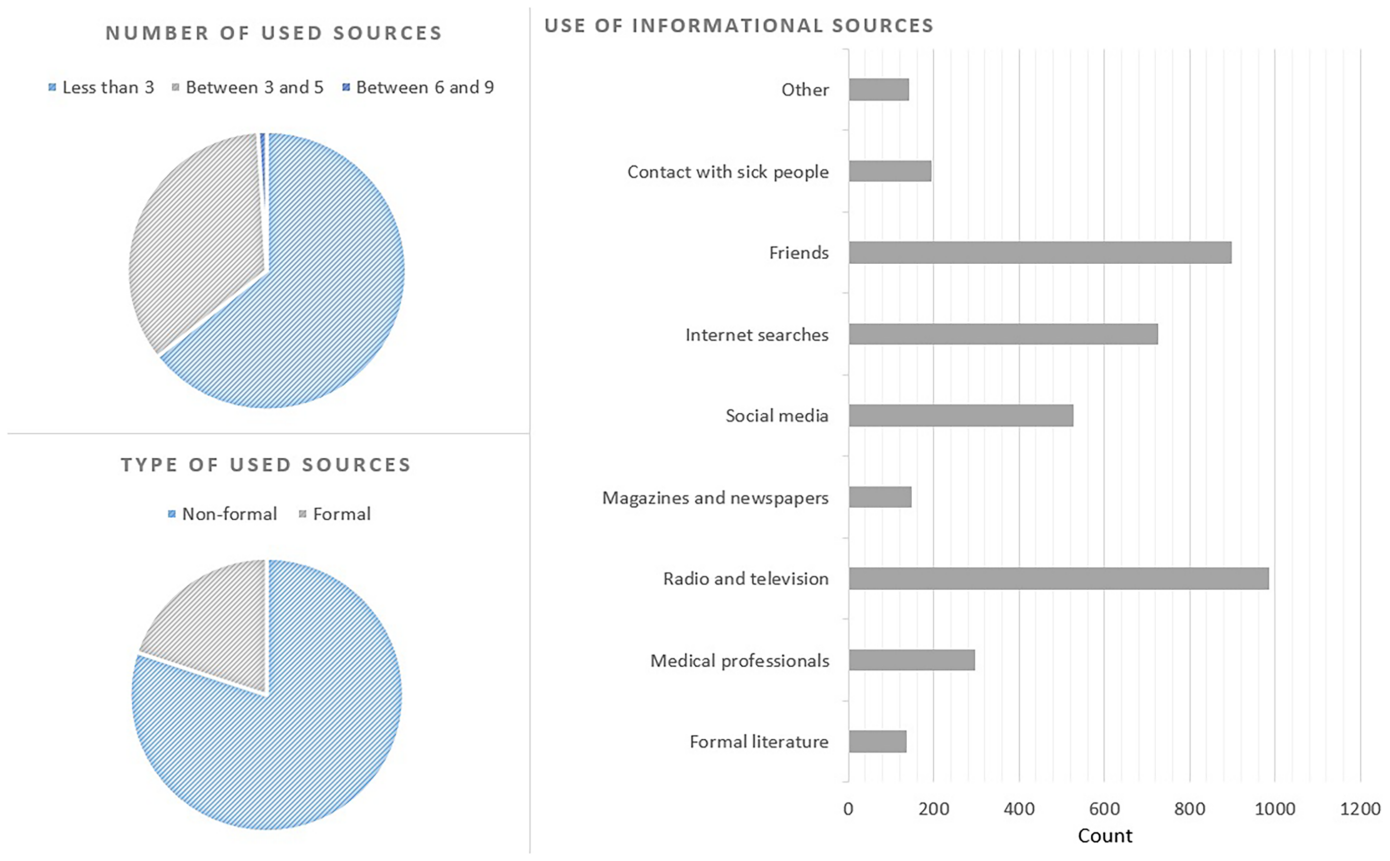
Usage of informational sources about dementia. Right: different informational sources versus the number of participants using them. Top left: proportions of participants using less than three, between three and five, and between six and nine informational sources. Bottom left: proportion of participants using formal informational sources (scientific literature, study books, lectures, and medical professionals) versus the proportion of participants using non‐formal sources (friends, conventional and social media, internet searches, etc.).

More than half of the participants reported drawing information about dementia from conventional media outlets such as radio, television, newspapers, and magazines (59.75%). A substantial proportion of the participants was receiving information from their immediate social circle (47.36%). Furthermore, 28% sourced information from social media and 38.34% from internet searches. About 16% received information from medical professionals and 7.23% informed themselves from scientific literature, lectures, or study books (Fig. 1). There were 1221 participants (64.40%) who reported using less than three sources of information about dementia and another 656 (34.60%) reported between three and five informational sources. Finally, 19 (1.00%) respondents reported using between six and nine informational sources. In terms of the quality of informational sources, 378 (19.90%) participants were receiving their information from formal literature and/or medical professionals (Fig. 1).

### Vignette recognition

**Figure 2:**
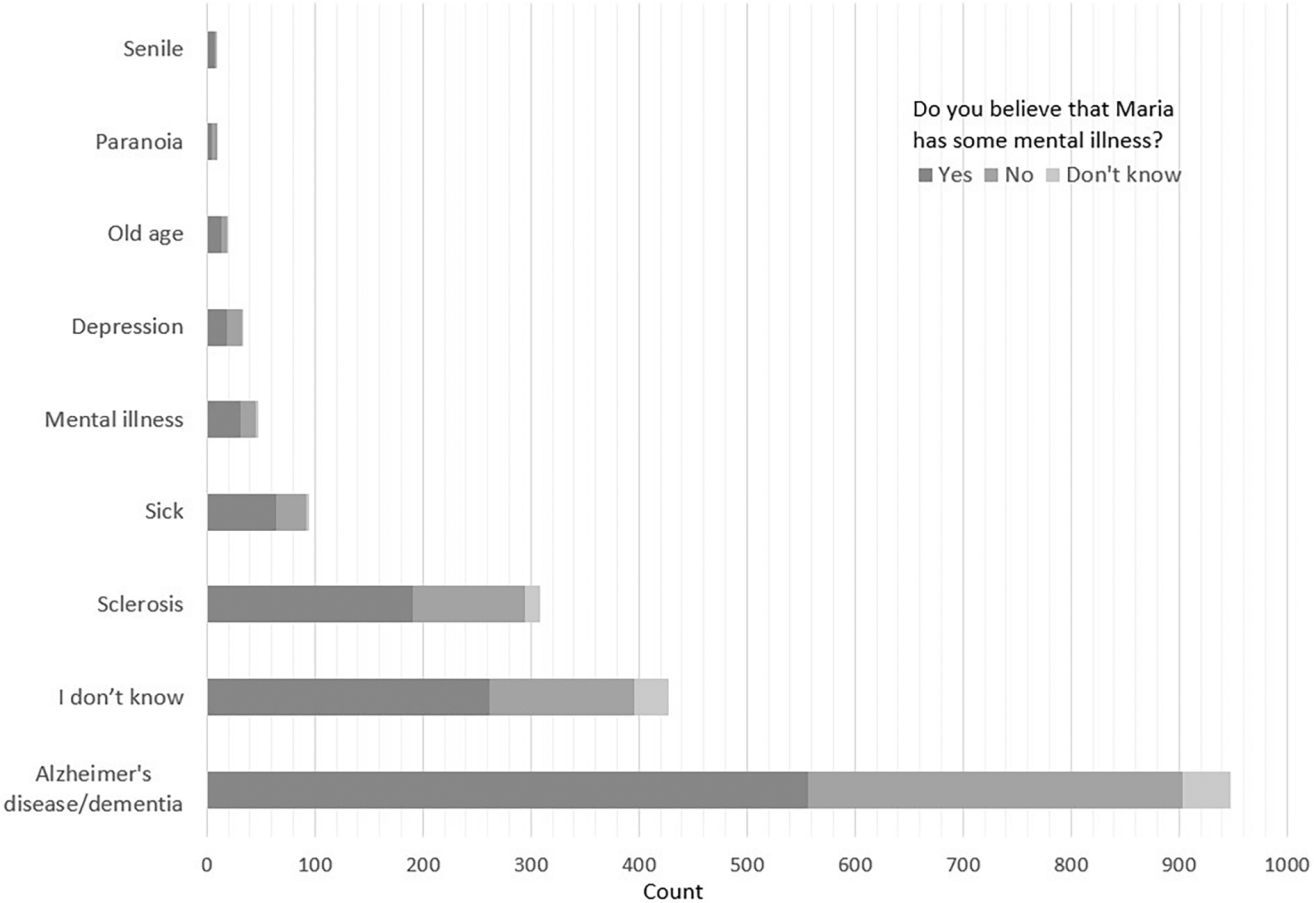
Summary of vignette recognition outcomes. Half of the participants recognised the described set of symptoms as dementia or Alzheimer's disease.

Overall, participants were able to recognise Maria's condition as abnormal with about 1% of the respondents accounting her symptoms to old age (Fig. 2). However, only half of the participants were able to correctly recognise the symptoms and identify the condition as Alzheimer's disease or dementia. Interestingly, about 16% of the respondents described Maria's condition as sclerosis, possibly referring to a leftover term from the socialist era, a shortened version of atherosclerotic dementia. However, over the years ‘sclerosis’ or ‘sclerotic’ have established themselves as derogatory terms often used in dismissive or insulting contexts. As Galina Goncharova and Teodora Karamelska write^60^:

‘*Even now*, *jokes and cartoons about grandparents with sclerosis circulate on Bulgarian internet platforms showing the tragicomedy of aging*. *We also remember stories about older people who would jump off balconies or who would become hopelessly lost in their home villages or even neighbourhoods; there were stories of people unable even to remember their own names*. “*Ignore them; they're sclerotic!*”*—this phrase was frequently used and affirmed* sclerosis *as an insult*, *a dismissive reference to the social stigma attached to the disease*’.

Having professional experience with dementia (*ꭓ* ^2^ = 6.016, degrees of freedom (df) = 1, *P* = 0.0142) and using a higher number of informational sources (*ꭓ* ^2^ = 8.1051, df = 2, *P* = 0.0173) were associated with successful vignette recognition. Complete results are available in Tables S1 *–* S3.

A little more than 60% of all participants believed that Maria was suffering from a mental illness. Interestingly, more than half of the respondents who identified Maria's condition as dementia or sclerosis also believed that she was mentally ill (Fig. 2). These findings demonstrate a persisting level of confusion surrounding mental illnesses and neurodegenerative disorders, possibly pointing to a much broader knowledge gap.

### Knowledge and beliefs about dementia

The average DKAS score for the sample was 9.51 points (SD = 4.66), with the highest score achieved by a respondent being 26 points out of 50 possible. More than half of the participants (56.7%) believed that dementia is a normal part of ageing and a similar proportion (54.1%) believed that recovering from dementia is possible (Table 2). While the overall performance was poor, the participants were somewhat more familiar with dementia care practices compared to other aspects of the disease (Table 2). There were 74.8% who did not believe that a healthy lifestyle reduces the risk of dementia and 51.6% did not recognise the importance of early diagnosis for improving a patient's quality of life. Another concerning finding was the high proportion of individuals who believed that it is impossible to communicate with a person who has advanced dementia (60.2%). The prevalence of such beliefs suggests that there might be a considerable amount of stigmatisation related to dementia. In line with this concern, about 76% of the respondents believed that people affected by advanced dementia do not respond to changes in their environment. Holding incorrect concepts of those affected by the disease may harm the quality of care and life, ultimately causing emotional turmoil for carers and patients. Therefore, addressing the spread of false beliefs about dementia is critically important, especially in domains with high social significance such as disease prevention and care.

**Table 2:** Summary of the results from the Dementia Knowledge Assessment Scale (DKAS) questionnaire

	False	Probably false	Don't know	Probably true	True
Causes and characteristics *n* (%) (mean = 1.79; SD = 1.40)					
1. Most forms of dementia do not generally shorten a person's life [F]	32 (1.7)	102 (5.4)	548 (28.9)	906 (47.8)	308 (16.2)
2. Blood vessel disease (vascular dementia) is the most common form of dementia [F]	42 (2.2)	233 (12.3)	949 (50.1)	535 (28.2)	137 (7.2)
3. People can recover from the most common forms of dementia [F]	49 (2.6)	121 (6.4)	702 (37.0)	659 (34.8)	365 (19.3)
4. Dementia is a normal part of the ageing process just like grey hairs [F]	14 (0.7)	69 (3.6)	738 (38.9)	894 (47.2)	181 (9.5)
5. Dementia does not result from physical changes in the brain [F]	7 (0.4)	147 (7.8)	868 (45.8)	789 (41.5)	85 (4.5)
6. Planning for end of life care is generally not necessary following a diagnosis of dementia [F]	26 (1.4)	119 (6.3)	751 (39.6)	838 (44.2)	162 (8.5)
7. Alzheimer's disease is the most common form of dementia [T]	3 (0.2)	19 (1.0)	371 (19.6)	741 (39.1)	762 (40.2)
Communication and behaviour *n* (%) (mean = 1.85; SD = 1.29)					
8. It is impossible to communicate with a person who has advanced dementia [F]	23 (1.2)	106 (5.6)	626 (33.0)	825 (43.5)	316 (16.7)
9. A person experiencing advanced dementia will not generally respond to changes in their physical environment [F]	2 (0.1)	50 (2.6)	402 (21.2)	836 (44.1)	606 (32.0)
10. It is important to correct a person with dementia when they are confused [F]	4 (0.2)	38 (2.0)	424 (22.4)	588 (31.0)	842 (44.0)
11. People experiencing advanced dementia often communicate through body language [T]	191 (10.1)	158 (8.3)	438 (23.1)	947 (49.9)	162 (8.5)
12. Uncharacteristic behaviours in a person experiencing dementia are generally a response to unmet needs [T]	9 (0.5)	76 (4.0)	474 (25.0)	947 (49.9)	390 (20.6)
13. Medications are the most effective way of treating behavioural symptoms of dementia [F]	9 (0.5)	236 (12.4)	864 (45.6)	552 (29.1)	235 (12.4)
Care considerations *n* (%) (mean = 4.09; SD = 2.34)					
14. People experiencing dementia do not generally have problems making decisions [F]	43 (2.3)	159 (8.4)	727 (38.3)	883 (46.6)	84 (4.4)
15. Movement is generally affected in the later stages of dementia [T]	23 (1.2)	76 (4.0)	401 (21.1)	692 (36.5)	704 (37.1)
16. Difficulty eating and drinking generally occurs in the later stages of dementia [T]	14 (0.7)	77 (4.1)	729 (38.4)	928 (48.9)	148 (7.8)
17. People with advanced dementia may have difficulty speaking [T]	23 (1.2)	91 (4.8)	595 (31.4)	941 (49.6)	246 (13.0)
18. People experiencing dementia often have difficulty learning new skills [T]	14 (0.7)	119 (6.3)	461 (24.3)	913 (48.2)	389 (20.5)
19. Daily care for a person with advanced dementia is effective when it focuses on providing comfort [T]	17 (0.9)	151 (8.0)	766 (40.4)	867 (45.7)	95 (5.0)
Risks and health promotion *n* (%) (mean = 1.78; SD = 1.41)					
20. Having high blood pressure increases a person's risk of developing dementia [T]	21 (1.1)	141 (7.4)	997 (52.6)	687 (36.2)	50 (2.6)
21. Maintaining a healthy lifestyle does not reduce the risk of developing the most common forms of dementia [F]	9 (0.5)	76 (4.0)	393 (20.7)	769 (40.6)	649 (34.2)
22. Symptoms of depression can be mistaken for symptoms of dementia [T]	50 (2.6)	268 (14.1)	810 (42.7)	660 (43.8)	108 (5.7)
23. The sudden onset of cognitive problems is characteristic of common forms of dementia [F]	70 (3.7)	230 (12.1)	746 (39.3)	788 (41.6)	62 (3.3)
24. Exercise is generally beneficial for people experiencing dementia [T]	235 (12.4)	291 (15.3)	636 (33.5)	642 (33.9)	92 (4.9)
25. Early diagnosis of dementia does not generally improve quality of life for people experiencing the condition [F]	53 (2.8)	323 (17.0)	542 (28.6)	753 (39.7)	225 (11.9)

Counts and proportions of answers are indicated for each item. The truthfulness of each item is marked with T (true) or F (false).

**Figure 3:**
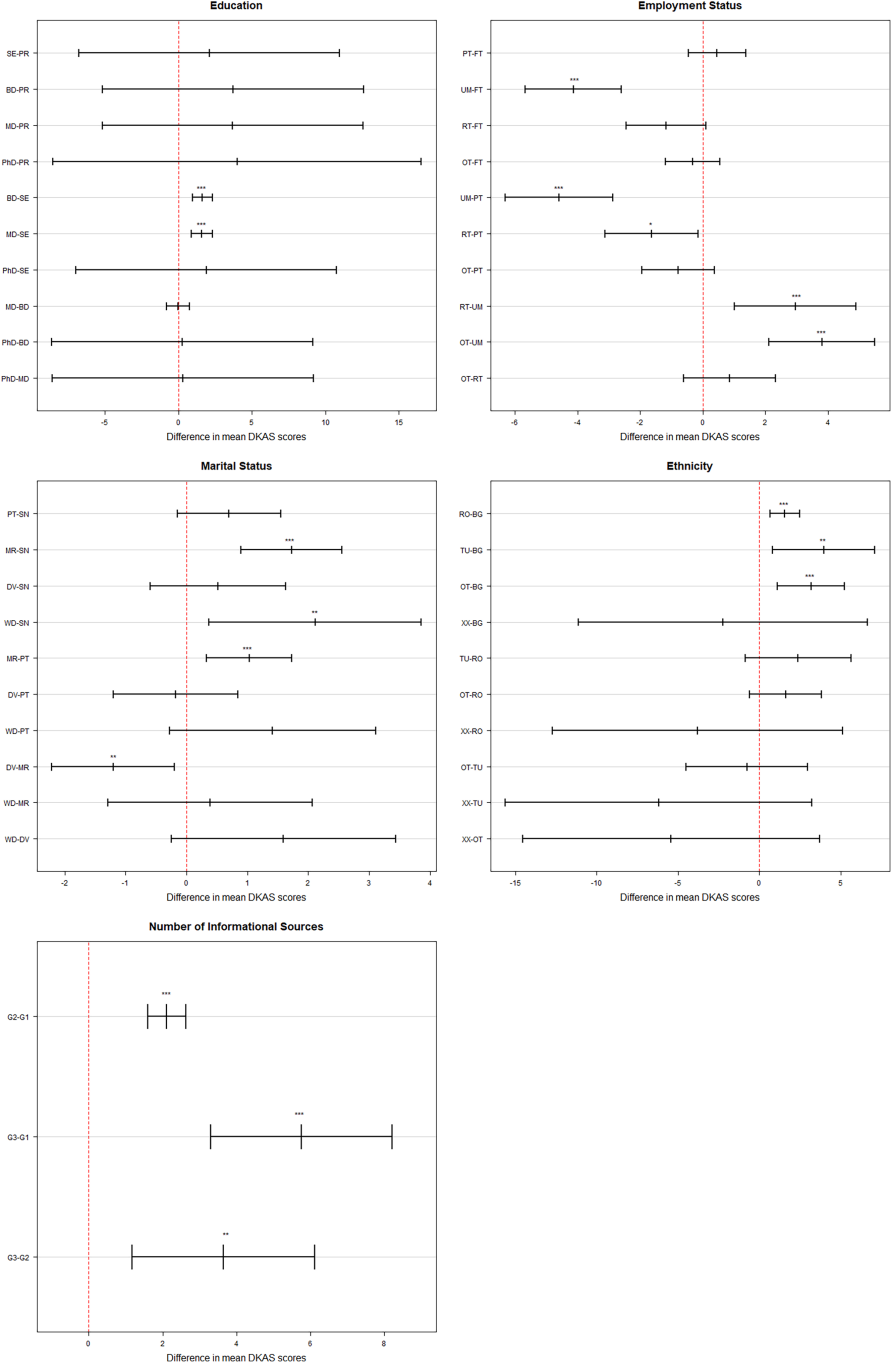
95% confidence intervals produced by Tukey's Honestly Significant Difference (HSD) pairwise comparisons. Results from the assessment of the differences in the mean Dementia Knowledge Assessment Scale (DKAS) scores between groups. **Education** (PR; primary; SC: secondary; BD: bachelor's degree; MD: master's degree), **Employment status** (FT: full‐time; PT: part‐time; UM: unemployed: RT, retired: OT, other), **Marital status** (SN: single; PT: partnership; MR: married; DV: divorced; WD: widowed), Ethnicity (BG, Bulgarian; RO, Roma; TU, Turkish; OT, other; XX, will not say), **Number of informational sources** (G1: less than 3; G2: between 3 and 5; G3: more than 5). Statistical significance is indicated as follows: *P < 0.05; **P < 0.01; ***P < 0.001.

Mean DKAS scores differed significantly in regard to education (*F*_4,891_ = 14.66, *P* < 0.001), employment status (*F*_4,1891_ = 15.81, *P* < 0.001), marital status (*F*_4,1891_ = 10.38, *P* < 0.001), ethnicity (*F*_4,1891_ = 12.17, *P* < 0.001) and quantity of used sources of information (*F*_8,1887_ = 29.71, *P* < 0.001). *Post hoc* Tukey tests revealed significant differences in DKAS scores of high school graduates compared to holders of bachelor's degree as well as holders of master's degree, at *P* < 0.001. Overall, a higher level of education was associated with higher DKAS scores (Fig. 3). On a similar note, unemployed individuals scored lower compared to those who were employed or retired. The pairwise analysis also revealed differences in mean DKAS scores of married individuals compared to singles (*P* < 0.001), individuals in a relationship (*P* < 0.001), and divorced (*P* < 0.01), with married individuals having higher DKAS scores compared to all other groups (Fig. 3). Curiously, Bulgarian ethnic background was associated with lower DKAS scores compared to Turkish (*P* < 0.001), Roma ethnic groups (*P* < 0.01), and those from other minority groups (*P* < 0.001).

Participants who reported having dementia in their family (*t* = 8.2162, df = 1179.94, *P* < 0.001), had experience as a caretaker (*t* = 6.1928, df = 488.99, *P* < 0.001), or whose profession required close contact with dementia patients (*t* = 6.5644, df = 76.03, *P* < 0.001) had higher levels of knowledge compared to those without such experiences. Finally, those using a higher number of informational sources (Fig. 3) and/or formal sources of information (*t* = 9.2503, df = 661.82, *P* < 0.001) had higher DKAS scores.

Those who successfully recognised the vignette had also higher DKAS scores (*t* = 4.7916, df = 1889.6, *P*‐value = *P* < 0.001), signalling that improved literacy may lead to a more accurate recognition of dementia manifestations. Complete results are available in Table S4.

### Recognition of dementia risk factors

**Figure 4:**
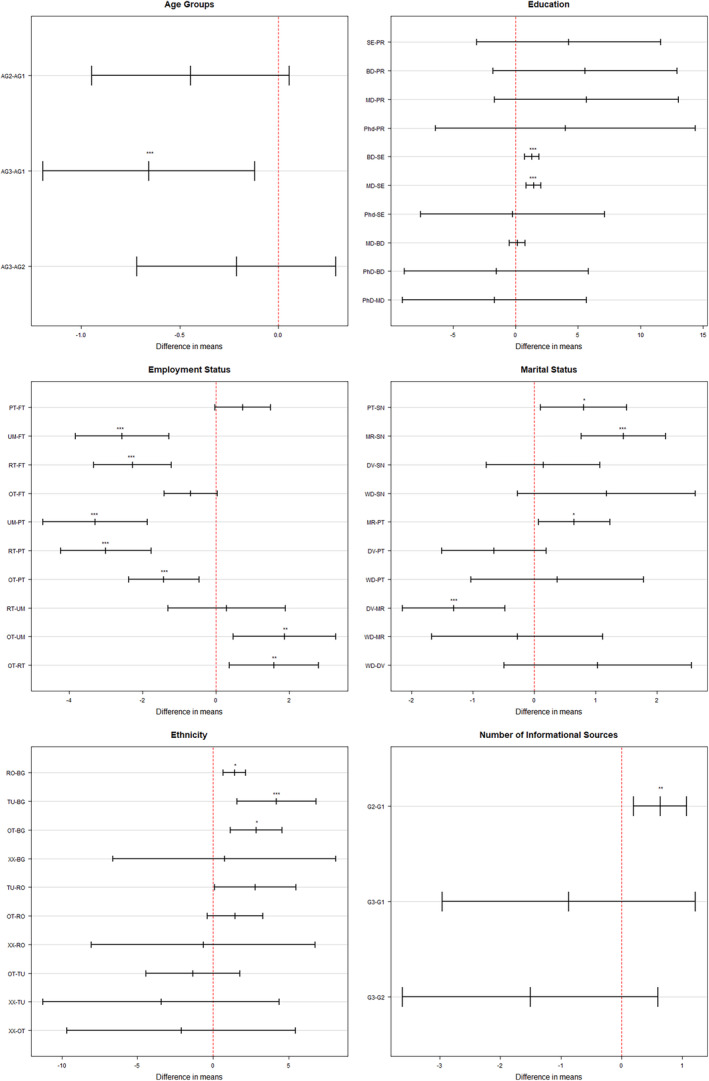
95% confidence intervals produced by Tukey's Honestly Significant Difference (HSD) pairwise comparisons. Results from the assessment of the differences in the mean number of correctly identified evidence‐based risk factors for dementia (answers ‘Contributes’ or ‘Probably contributes’ were considered as correct identification). **Age groups** (AG1: 18–35 years; AG2: 36–54 years; AG3: 55+), **Education** (PR: primary; SC: secondary; BD: bachelor's degree; MD: master's degree), **Employment status** (FT: full time; PT: part‐time; UM: unemployed; RT: retired; OT: other), **Marital status** (SN: single; PT: partnership; MR: married; DV: divorced; WD: widowed), Ethnicity (BG: Bulgarian; RO: Roma; TU: Turkish; OT: other; XX: will not say), Number of informational Sources (G1: less than 3; G2: between 3 and 5; G3: more than 5).

Nearly 30% of the participants were able to identify old age and genetics as contributors to the development of dementia (Table 3). Among the least recognised modifiable risk factors were lack of physical activity (14.00%), smoking (25.00%), depression (30.00%), lack of cognitive activity (31.00%), stroke (33.00%), and social isolation (35.00%). Moderately known factors were, high alcohol consumption (72.00%), unhealthy diet (70.00%), diabetes (68.00%), high blood pressure (63.00%), deafness (59.00%), cardiovascular disease (57.00%), and head trauma (54.00%). The mean number of correctly identified risk factors was 7.5 (SD = 3.9), with only 0.3% of the participants identifying all 17 risk factors. Significant group differences were found in age (*F*_2,1893_ = 4.34, *P* = 0.013), education (*F*_4,1891_ = 15.90, *P* < 0.001), employment status (*F*_4,1891_ = 19.64, *P* < 0.001), marital status (*F*_4,1891_ = 10.44, *P* < 0.001) ethnicity (*F*_4,1891_ = 15.42, *P* < 0.001) and quantity of informational sources (*F*_2,1893_ = 6.5687, *P* < 0.001). Pairwise comparisons further revealed that the youngest age group recognised more dementia risk factors than the oldest (*P* < 0.001, Fig. 4). Similarly, unemployed and retired individuals were less successful in identifying dementia risk factors compared to employed individuals (*P* < 0.001), and single and divorced individuals had a worse performance compared to those in relationships (*P* < 0.001). Ethnic Bulgarians had a lower number of identified risk factors compared to individuals from the Turkish, Roma, or other ethnic groups (Fig. 4). From the experiential factors only professional experience with dementia patients was associated with a higher number of correctly identified true dementia risk factors (*t* = 4.1322, df = 77.32, *P* < 0.001). Finally, those using formal informational sources were able to identify more dementia risk factors (*t* = −4.1058, df = 677.88, *P* < 0.001). Complete results are available in Table S5 from the Supplementary Material.

**Table 3:** Results from binomial tests of true dementia risk factors

Item	Answer categories	*n*	Estimated proportions	*P*‐value	Direction of effect
Non‐modifiable risk factors					
Genetic predisposition	Contributes	568	0.30	<0.001	Incorrect
	Does not contribute	1328	0.70		
Old age	Contributes	515	0.27	<0.001	Incorrect
	Does not contribute	1381	0.73		
Modifiable risk factors					
High alcohol consumption	Contributes	1356	0.72	<0.001	Correct
	Does not contribute	540	0.28		
Stroke	Contributes	633	0.33	<0.001	Incorrect
	Does not contribute	1263	0.77		
Head trauma	Contributes	1021	0.54	<0.001	Correct
	Does not contribute	875	0.46		
Unhealthy diet	Contributes	1318	0.70	<0.001	Correct
	Does not contribute	578	0.30		
Diabetes	Contributes	1288	0.68	<0.001	Correct
	Does not contribute	608	0.32		
High blood pressure	Contributes	1189	0.63	<0.001	Correct
	Does not contribute	707	0.37		
Hearing loss	Contributes	1116	0.59	<0.001	Correct
	Does not contribute	780	0.41		
Cardiovascular disease	Contributes	1077	0.57	<0.001	Correct
	Does not contribute	819	0.43		
Obesity	Contributes	809	0.43	<0.001	Incorrect
	Does not contribute	1087	0.57		
Fewer years of education	Contributes	794	0.42	<0.001	Incorrect
	Does not contribute	1102	0.58		
Social isolation	Contributes	666	0.35	<0.001	Incorrect
	Does not contribute	1230	0.65		
Lack of cognitive activity	Contributes	589	0.31	<0.001	Incorrect
	Does not contribute	1307	0.69		
Depression	Contributes	560	0.30	<0.001	Incorrect
	Does not contribute	1336	0.70		
Smoking	Contributes	469	0.25	<0.001	Incorrect
	Does not contribute	1427	0.75		
Lack of physical activity	Contributes	261	0.14	<0.001	Incorrect
	Does not contribute	1635	0.86		

Estimated proportions for ‘Contributes’ answers were tested against chance (0.50). *P* < 0.05 signify higher than chance probabilities for answering ‘Contributes’ to a true risk factor item. Answer categories were formulated as follows: Contributes = the sum of all ‘contributes’ and ‘probably contributes’ answers. Does not contribute = the sum of all ‘does not contribute’ and ‘probably does not contribute’ answers plus all neutral answers.

More than half of the participants considered viral and bacterial infections contributors to dementia – 58.00% and 61.00% respectively. Parasitic infection, on the other hand, was considered a contributor to dementia by 41% of the respondents (Table S6).

An alarmingly low number of participants correctly identified the sham factors as non‐contributors to dementia. A common belief was that amalgam dental fillings, stress, and laziness are involved in the genesis of dementia, with respective proportions of ‘Contributes’ answers 0.61, 0.58, and 0.58, all significantly higher than chance, *P* < 0.001. Similarly, 40% marked witchcraft as a contributor to dementia, and between 32% and 37% believed that vaccines, fate, and aspartame may contribute to the onset of dementia (Table 4). Such observations demonstrate that the spread of false beliefs, based on popular culture, is still a deeply rooted issue within the Bulgarian society that may endure debunking and factual education.

**Table 4:** Results from binomial tests for items without scientific evidence

Item	Answer categories	*n*	Estimated proportions	*P*‐value	Direction of effect
Dental fillings	Does not contribute	178	0.09	1.00	Incorrect
	Contributes	1253	0.67		
	Do not know	465	0.24		
Laziness	Does not contribute	251	0.13	1.00	Incorrect
	Contributes	1108	0.58		
	Do not know	537	0.29		
Stress	Does not contribute	185	0.10	1.00	Incorrect
	Contributes	1094	0.58		
	Do not know	617	0.32		
Aluminium	Does not contribute	341	0.18	1.00	Incorrect
	Contributes	915	0.48		
	Do not know	640	0.34		
Curse/witchcraft	Does not contribute	633	0.33	1.00	Incorrect
	Contributes	749	0.40		
	Do not know	514	0.27		
Aspartame	Does not contribute	340	0.18	1.00	Incorrect
	Contributes	697	0.37		
	Do not know	859	0.45		
Fate	Does not contribute	342	0.18	1.00	Incorrect
	Contributes	686	0.36		
	Do not know	868	0.46		
Vaccines	Does not contribute	369	0.19	1.00	Incorrect
	Contributes	602	0.32		
	Do not know	925	0.49		
Poverty	Does not contribute	676	0.36	1.00	Incorrect
	Contributes	297	0.16		
	Do not know	923	0.48		
Weak will	Does not contribute	864	0.46	0.099	Incorrect
	Contributes	216	0.11		
	Do not know	816	0.43		

Estimated proportions for ‘Does not contribute’ answers were tested against chance (0.50). *P* < 0.05 signifies higher than chance probabilities of answering ‘Does not contribute’ for a particular item. Answer categories were formulated as follows: Contributes = the sum of all ‘contributes’ and ‘probably contributes’ answers. Does not contribute = the sum of all ‘does not contribute’ and ‘probably does not contribute’.

## DISCUSSION

This study is the first to assess dementia knowledge in the Bulgarian general population. Poor knowledge, limited risk factors awareness, and consistent presence of misconceptions were among the main findings of this study. Gaps in knowledge were expected to a degree, as dementia is still not recognised as a health priority in Bulgaria, resulting in a limited outreach on the topic and a lack of coordinated educational efforts. However, this study provided compelling evidence for the extent of misinformation and illiteracy occurring in Bulgaria.

### Health literacy in Bulgaria

Our results demonstrated alarmingly low levels of dementia knowledge among Bulgarian society, with the mean DKAS of our sample being well below the averages reported for the United States, India, Europe, South/Central America, Canada, Asia, and Africa.^61^ This trend is consistently confirmed when comparing our sample (mean = 9.51; SD = 4.66) to general samples from Saudi Arabia (mean = 20.50; SD = 9.30),^62^ Switzerland and Italy (mean = 22.43; SD = 8.88),^63^ Belgium (mean = 23.65; SD = 7.39)^64^ and Australia (mean = 27.72; SD = 9.15).^65^ This compelling evidence strongly indicates that Bulgaria is lagging in terms of dementia literacy. However, it is likely that poor health knowledge in Bulgaria is not limited to dementia. In line with this, a previous study estimated that approximately 1/3 of Bulgarian citizens have a poor level of health literacy.^66^ Furthermore, a comparative analysis of the European Health Literacy Survey (HLS‐EU), reported Bulgaria having the lowest health literacy index among the examined European countries.^67^ Thus, our results confirm the unsatisfactory levels of health literacy in Bulgaria, by unveiling significant gaps in dementia knowledge.

### Recognising dementia symptoms

The vignette, used in the present work, described moderate symptoms of Alzheimer's disease, presenting the participants with well‐defined disease manifestations. However, in reality, Alzheimer's disease is characterised by a gradual onset, making recognition more difficult, especially in the early stages of the disease. Thus, it might be the case that identifying dementia in real life is more difficult compared to a vignette task, resulting in even lower rates of identification. Second, in contrast to other populations, our sample had a poor performance on the vignette task. For instance, 82% of 2000 community‐dwelling Australian adults identified the same vignette as ‘dementia’ or ‘Alzheimer's disease’.^31^ Similarly, a study exploring dementia knowledge among Australian minorities reported that third‐generation Australians (85%) were more likely to recognise dementia symptoms in an identical vignette compared to Italian (61%), Greek (58%), and Chinese (72%) Australians.^32^ Interestingly, our results (50%) are close to the recognition rate of Greek Australians, suggesting impoverished knowledge about dementia symptoms may be prominent not only in Bulgaria but in the Balkan region. Despite the chance recognition of the vignette, there is an encouraging aspect of our results as almost all participants were able to identify Maria's condition as abnormal. Thus, we believe that observing similar behaviour in real life will cause health concerns and trigger help‐seeking actions despite the inability to identify the disease.

### Knowledge about risk factors

We found a consistent lack of awareness about dementia risk factors, constituting a lack of recognition of the protective role of personal prevention. Considering that about 40% of the worldwide cases of dementia are due to modifiable risk factors and are considered preventable,^36^ awareness campaigns should emphasise the protective role and the importance of a healthy lifestyle, especially for individuals with a family history of dementia. Interestingly, cardiovascular disease, diabetes, and high blood pressure were acknowledged by a considerable number of participants, marking a contrast between our results and previous studies, reporting cardiovascular factors as less recognised contributors to dementia.^21^, ^23^ This might be explained by the emphasis on the management of cardiovascular diseases in Bulgaria. Currently, circulatory diseases are the leading cause of death in Bulgaria, with rates being well above the average for the European Union.^68^ Thus, Bulgarians may be more aware of the implications of cardiovascular diseases, including the risk of developing dementia. The fact that depression and social isolation were mostly unknown as risk factors for dementia bears great social significance as elderly individuals in rural regions of Bulgaria face a high risk of social isolation due to outmigration or immigration. Family members and policy‐makers should consider this connection and create an environment of support and care for those spending their late years in remote regions. A possible solution is the development of support groups, hobby clubs, and social centres for elders. Creating a proactive social environment can reduce the sense of isolation, replacing it with a sense of purpose and community.

### Misconceptions about dementia

Our study identified a number of misconceptions about dementia. For instance, more than half of the participants believed dementia was a normal part of ageing and that recovery was possible. Several review articles reported similar findings, concluding these misconceptions are among the most commonly occurring false beliefs about dementia.^20^, ^21^ Many of the revealed misconceptions related to the causes of the disease, particularly beliefs that amalgam dental fillings, laziness, and stress contribute to the onset of dementia. Interestingly, a considerable proportion of participants (about 40%) placed fate and witchcraft among the factors causing dementia. Previous works have also reported supernatural or theological explanations for dementia. For instance, in South Asian populations dementia was seen as God's punishment, or demons.^22^ Similarly, nearly 30% of the respondents in a study from Brazil stated that evil eye and/or fate were causal factors for dementia.^33^ As superstitious explanations of dementia may contribute to stigmatisation and deepen feelings of helplessness in relatives and family carers, it is important to create an environment where fictional beliefs are refuted through evidence. Strategies for broad dissemination of scientific findings may help in filtering evidence‐based information from opinions, speculations, and non‐scientific theories.

### Ethnic disparities in knowledge

In contrast to previous studies,^20^, ^32^, ^61^ our results suggest that minority groups are more knowledgeable about dementia and risk factors compared to ethnic Bulgarians. This may be partially explained by the choice of an online survey methodology. The Roma minority group is among the most deprived and facing unequal access to employment, education, housing, and health. According to the National Statistical Institute of Bulgaria, poverty is most prevalent among the Roma ethnic group, with 63.3% being below or at the poverty line.^69^ Therefore, using an online survey as an acquisition method may have posed an implicit bias toward more educated and financially stable individuals. Additionally, ethnic groups represent a small proportion of our data, thus while our results are valid within the sample it is difficult to draw general conclusions. Nevertheless, to the best of our knowledge, this is the first study to consider dementia literacy among ethnic communities in Bulgaria. While the results are inconclusive, they unveil an intriguing perspective that warrants further research.

### Implications

Our study highlighted significant misconceptions about the factors contributing to dementia, alongside a limited capacity to recognise its actual risk factors. These findings reflect the inadequate state of primary prevention for dementia within the Bulgarian population. Notably, despite the World Health Organization's 2017 action plan encouraging countries to launch campaigns aimed at improving dementia awareness,^70^ Bulgaria seems to have made little progress in adopting these recommendations nearly a decade later. Despite the delayed response, the results from this study can be used to inform public awareness initiatives designed to address the specific knowledge gaps identified here. Furthermore, having particular knowledge about the prevailing misconceptions allows the implementation of multiple strategies against the spread of false information, including debunking and pre‐bunking techniques. On a similar note, some of the identified misconceptions unveil underlying stigmatic beliefs about the disease and the individuals affected by the condition. To create a safe and inclusive environment, ageist stereotypes and negative framings of the condition should be actively addressed.

Our findings also revealed gaps in knowledge related to providing care and effectively communicating with individuals who are ill. The identified misconceptions can be used as a starting point for creating community programs, directed toward family members involved in caretaking. The results from the last wave of the European Values Study showed that 77% of Bulgarians believe that caring for their parents is their responsibility.^60^ Consequently, many individuals undertake the demanding responsibility of serving as full time caregivers for their loved ones. However, a significant contributor to the emotional strain and frustration experienced by these caregivers is their limited understanding of the behaviours exhibited by dementia patients and their lack of knowledge about effective care strategies. Therefore, the establishment of training and support programs tailored for non‐professional caregivers would provide substantial benefits to affected families. Such initiatives would foster a more supportive and resource‐equipped environment for caregivers, ultimately enhancing the overall quality of life for dementia patients.

Finally, while our results are specific to the state of dementia knowledge in Bulgaria, we believe that countries with similar dementia response profiles can also utilise them to their benefit. For instance, according to the 2023 European Dementia Monitor report,^5^ North Macedonia, Serbia, Armenia, Ukraine, and Romania have similar ratings to Bulgaria in terms of their response to dementia. Considering the cultural, historical, and policy‐based similarities with Bulgaria, dementia literacy in these countries may exhibit similar deficiencies to the ones observed in Bulgaria. Therefore, our findings have the potential to contribute to improving dementia awareness not only in Bulgaria but also across the broader Balkan and Black Sea regions.

### Limitations

Several limitations should be considered in the interpretation of our results. First, since all respondents were volunteers, sourced by the contracted agency, our study is prone to selection bias as it represents those who were in the database and willing to complete the survey. Similarly, using an online survey introduces another level of selection bias as it reaches only individuals who are actively using internet technologies. Therefore, it is possible that some groups of the population were underrepresented, especially older individuals and those who do not use computers in their daily lives. Last, this study used a quantitative approach to examine dementia knowledge in Bulgaria. Future research may benefit from supplementing quantitative data with narrative or phenomenological approaches that can provide insight into personal experiences with dementia.

## CONCLUSIONS

We found severe gaps in dementia knowledge and a widespread presence of false beliefs among the Bulgarian population. Thus, we encourage Bulgarian policy‐makers to reconsider the importance of dementia as a social issue and take coordinated actions to educate society and eradicate harmful misconceptions.

## AUTHOR CONTRIBUTIONS

Conceptualisation, S.L. Methodology, S.L. Investigation, S.L. Writing – original draft preparation, S.L. Writing – review and editing, S.L and D.P‐A. Supervision, D.P‐A. Project administration, D.P‐A. Funding acquisition, D.P‐A.

## DISCLOSURE

None of the authors have a conflict of interest to disclose.

## Supporting information


**Table S1.** Results from *χ* ^2^ tests of independence assessing the connection between vignette recognition ability and sociodemographic factors, previous experience with dementia, and number and quality of used informational sources. The results show that the only factors associated with the ability to recognise the symptoms of dementia from a vignette are having professional contact with dementia patients and the number of used informational sources.
**Table S2.** Extended contingency table for outcomes of vignette recognition task and having professional contact with dementia patients. Results from the *post hoc* analysis of Pearson's *χ* ^2^ test residuals are included in the bottom section of the table. Statistical significance is indicated as follows: *P < 0.05; **P < 0.01; ***P < 0.001.
**Table S3.** Extended contingency table for outcomes of vignette recognition task and the number of used informational sources about dementia. Results from the *post hoc* analysis of Pearson's *χ* ^2^ test residuals are included in the bottom section of the table. Statistical significance is indicated as follows: *P < 0.05; **P < 0.01; ***P < 0.001.
**Table S4.** Group differences in Dementia Knowledge Assessment Scale (DKAS) scores. Groups are formulated on the basis of sociodemographic characteristics, previous experience with dementia, used informational sources about dementia, and vignette recognition.
**Table S5.** Results from analyses of recognised dementia risk factors in terms of sociodemographic characteristics, previous experience with dementia, and used informational sources about dementia.
**Table S6.** Results from two‐tailed binomial tests for items with emerging evidence.

## Data Availability

The data that support the findings of this study are available on request from the corresponding author. The data are not publicly available due to privacy or ethical restrictions.

## References

[1] Duong S , Patel T , Chang F . Dementia: what pharmacists need to know. Can Pharm J 2017; 150: 118–129. 10.1177/1715163517690745. PMC5384525 28405256

[2] Livingston G , Huntley J , Liu KY *et al*. Dementia prevention, intervention, and care: 2024 report of the lancet standing commission. Lancet 2024; 404: 572–628. 10.1016/S0140-6736(24)01296-0. 39096926

[3] Georges J , Miller O , Bintener C . Estimating the prevalence of dementia in Europe. Luxembourg: Alzheimer Europe, 2020. 10.13140/RG.2.2.16880.81923.

[4] Pitheckoff N . Aging in the Republic of Bulgaria. Gerontologist 2017; 57: 809–815. 10.1093/geront/gnx075. 28931119

[5] Alzheimer Europe . European Dementia Monitor 2023 ‐ Comparing and Benchmarking National Dementia Strategies and Policies. Luxembourg: Alzheimer Europe, 2023.

[6] Lo R . Uncertainty and health literacy in dementia care. Tzu Chi Med J 2020; 32: 14–18. 10.4103/tcmj.tcmj_116_19. 32110514 PMC7015016

[7] Oliveira D , Bosco A , Di Lorito C . Is poor health literacy a risk factor for dementia in older adults? Systematic literature review of prospective cohort studies. Maturitas 2019; 124: 8–14. 10.1016/j.maturitas.2019.03.010. 31097184

[8] Arce Rentería M , Vonk JMJ , Felix G *et al*. Illiteracy, dementia risk, and cognitive trajectories among older adults with low education. Neurology 2019; 93: e2247–e2256. 10.1212/WNL.0000000000008587. 31722961 PMC6937498

[9] Kaup AR , Simonsick EM , Harris TB *et al*. Older adults with limited literacy are at increased risk for likely dementia. J Gerontol A Biol Sci Med Sci 2014; 69: 900–906. 10.1093/gerona/glt176. 24158765 PMC4067115

[10] Scazufca M , Almeida OP , Menezes PR . The role of literacy, occupation and income in dementia prevention: the São Paulo Ageing & Health Study (SPAH). Int Psychogeriatr 2010; 22: 1209–1215. 10.1017/S1041610210001213. 20678301

[11] Chang C‐Y , Hsu H‐C . Relationship between knowledge and types of attitudes towards people living with dementia. Int J Environ Res Public Health 2020; 17: 3777. 10.3390/ijerph17113777. 32466533 PMC7312095

[12] Li Y‐T , Bai J‐X , He J‐M , Yang S‐W , Huang H‐L . The mediating role of attitudes towards dementia on the relationship between dementia knowledge and behaviors towards persons with dementia: a cross‐sectional study. J Multidiscip Healthc 2023; 16: 4213–4225. 10.2147/JMDH.S443189. 38156291 PMC10752817

[13] Lea E , Robinson A , Doherty K . Relationship between dementia knowledge and occupational strain among staff of residential facilities for older adults: a cross‐sectional survey. Ageing Int 2023; 48: 1221–1237. 10.1007/s12126-023-09523-y.

[14] Teichmann B , Gkioka M , Kruse A , Tsolaki M . Informal Caregivers' attitude toward dementia: the impact of dementia knowledge, confidence in dementia care, and the behavioral and psychological symptoms of the person with dementia. A cross‐sectional study. J Alzheimers Dis 2022; 88: 971–984. 10.3233/JAD-215731. 35723101 PMC9484115

[15] Rasmussen BM , Andersen PT , Waldorff FB , Berg‐Beckhoff G . Effectiveness of dementia education for professional care staff and factors influencing staff‐related outcomes: an overview of systematic reviews. Int J Nurs Stud 2023; 142: 104469. 10.1016/j.ijnurstu.2023.104469. 37080121

[16] Yang T , Huang Y , Li X *et al*. Knowledge, attitudes, and stigma related to dementia among illiterate and literate older adults in Shanghai. Risk Manag Healthc Policy 2021; 14: 959–966. 10.2147/RMHP.S296044. 33727872 PMC7955023

[17] Herrmann LK , Welter E , Leverenz J *et al*. A systematic review of dementia‐related stigma research: can we move the stigma dial? Am J Geriatr Psychiatry 2018; 26: 316–331. 10.1016/j.jagp.2017.09.006. 29426607

[18] Werner P , Goldstein D , Karpas DS , Chan L , Lai C . Help‐seeking for dementia: a systematic review of the literature. Alzheimers Dement 2014; 28: 299–310. 10.1097/WAD.0000000000000065. 25321607

[19] Zhang H , Zhou Y , Ma J , Li Z . Understanding help‐seeking decisions in people with subjective cognitive decline: a systematic review of qualitative studies. Geriatr Nurs 2021; 42: 1507–1516. 10.1016/j.gerinurse.2021.10.013. 34735997

[20] Cahill S , Pierce M , Werner P , Darley A , Bobersky A . A systematic review of the Public's knowledge and understanding of Alzheimer's disease and dementia. Alzheimers Dement 2015; 29: 255–275. 10.1097/WAD.0000000000000102. 26207322

[21] Cations M , Radisic G , Crotty M , Laver KE . What does the general public understand about prevention and treatment of dementia? A systematic review of population‐based surveys. PLoS One 2018; 13: e0196085. 10.1371/journal.pone.0196085. 29672559 PMC5908164

[22] Hossain MZ , Stores R , Hakak Y , Crossland J , Dewey A . Dementia knowledge and attitudes of the general public among the Bangladeshi Community in England: a focus group study. Dement Geriatr Cogn Disord 2019; 48: 290–296. 10.1159/000506123. 32213774

[23] Zülke A , Luppa M , Köhler S , Riedel‐Heller SG . Was weiß die Bevölkerung über Risiko‐ und Schutzfaktoren für Demenz? Eine internationale Übersicht zum aktuellen Kenntnisstand in verschiedenen Ländern. Nervenarzt 2023; 94: 384–391. 10.1007/s00115-023-01471-x. 37099170

[24] Zheng Y‐B , Shi L , Gong Y‐M *et al*. Public awareness and knowledge of factors associated with dementia in China. BMC Public Health 2020; 20: 1567. 10.1186/s12889-020-09665-7. 33069235 PMC7568826

[25] Song D , Yu D , Sun Q . Perception and knowledge of dementia prevention and its associated socio‐demographic factors in China: a community‐based cross‐sectional study. Front Neurosci 2022; 16: 1093169. 10.3389/fnins.2022.1093169. 36545535 PMC9760739

[26] Skowronek A , Bojkowska‐Otrębska K , Łabuz‐Roszak B . Public knowledge about dementia in Poland—a survey study. J Clin Med 2023; 12: 7675. 10.3390/jcm12247675. 38137744 PMC10743585

[27] Kjelvik G , Rokstad AMM , Stuebs J *et al*. Public knowledge about dementia risk reduction in Norway. BMC Public Health 2022; 22: 2046. 10.1186/s12889-022-14433-w. 36348300 PMC9644554

[28] Stojic J , Petrosanec M , Milosevic M , Boban M . The attitude and knowledge of medical students regarding dementia. Acta Neurol Belg 2022; 122: 625–630. 10.1007/s13760-022-01939-8. 35429287

[29] Tsolaki M , Paraskevi S , Degleris N , Karamavrou S . Attitudes and perceptions regarding Alzheimer's disease in Greece. Am J Alzheimers Dis Demen 2009; 24: 21–26. 10.1177/1533317508325990. PMC10846124 19047472

[30] Annear MJ , Toye C , Elliott K‐EJ , McInerney F , Eccleston C , Robinson A . Dementia knowledge assessment scale (DKAS): confirmatory factor analysis and comparative subscale scores among an international cohort. BMC Geriatr 2017; 17: 168. 10.1186/s12877-017-0552-y. 28760154 PMC5537989

[31] Low L , Anstey KJ . Dementia literacy: recognition and beliefs on dementia of the Australian public. Alzheimers Dement 2009; 5: 43–49. 10.1016/j.jalz.2008.03.011. 19118808

[32] Low L‐F , Anstey KJ , Lackersteen SM *et al*. Recognition, attitudes and causal beliefs regarding dementia in Italian, Greek and Chinese Australians. Dement Geriatr Cogn Disord 2010; 30: 499–508. 10.1159/000321667. 21252544

[33] Blay SL , Piza Peluso É d T . The Public's ability to recognize Alzheimer disease and their beliefs about its causes. Alzheimers Dement 2008; 22: 79–85. 10.1097/WAD.0b013e31815ccd47. 18317251

[34] Malik YK , Ray A , Singh S , Gupta R . Dementia literacy and familiarity with term dementia: an exploratory study from a psychiatry outpatient setting. J Neurosci Rural Pract 2023; 15: 227–232. 10.25259/JNRP_475_2023. 38746503 PMC11090581

[35] Annear MJ , Toye CM , Eccleston CE *et al*. Dementia knowledge assessment scale: development and preliminary psychometric properties. J Am Geriatr Soc 2015; 63: 2375–2381. 10.1111/jgs.13707. 26503020

[36] Livingston G , Huntley J , Sommerlad A *et al*. Dementia prevention, intervention, and care: 2020 report of the lancet commission. Lancet 2020; 396: 413–446. 10.1016/S0140-6736(20)30367-6. 32738937 PMC7392084

[37] Saeed A , Lopez O , Cohen A , Reis SE . Cardiovascular disease and Alzheimer's disease: the heart–brain Axis. J Am Heart Assoc 2023; 12: e030780. 10.1161/JAHA.123.030780. 37929715 PMC10727398

[38] Justin BN , Turek M , Hakim AM . Heart disease as a risk factor for dementia. Clin Epidemiol 2013; 5: 135–145. 10.2147/CLEP.S30621. 23658499 PMC3641811

[39] Cho S , Yang P‐S , Kim D *et al*. Association of cardiovascular health with the risk of dementia in older adults. Sci Rep 2022; 12: 15673. 10.1038/s41598-022-20072-3. 36123419 PMC9485258

[40] Kuźma E , Lourida I , Moore SF , Levine DA , Ukoumunne OC , Llewellyn DJ . Stroke and dementia risk: a systematic review and meta‐analysis. Alzheimers Dement 2018; 14: 1416–1426. 10.1016/j.jalz.2018.06.3061. 30177276 PMC6231970

[41] Pendlebury ST , Rothwell PM . Incidence and prevalence of dementia associated with transient ischaemic attack and stroke: analysis of the population‐based Oxford vascular study. Lancet Neurol 2019; 18: 248–258. 10.1016/S1474-4422(18)30442-3. 30784556 PMC6390174

[42] Xu Lou I , Ali K , Chen Q . Effect of nutrition in Alzheimer's disease: a systematic review. Front Neurosci 2023; 17: 1147177. 10.3389/fnins.2023.1147177. 37214392 PMC10194838

[43] Nicoli C , Galbussera AA , Bosetti C *et al*. The role of diet on the risk of dementia in the oldest old: the Monzino 80‐plus population‐based study. Clin Nutr 2021; 40: 4783–4791. 10.1016/j.clnu.2021.06.016. 34242918

[44] Guerchet M , Prina M , Prince M , Albanese E , Siervo M , Acosta D . Nutrition and Dementia‐A Review of Available Research. London: Alzheimer's Disease International (ADI), 2014.

[45] Baumgart M , Snyder HM , Carrillo MC , Fazio S , Kim H , Johns H . Summary of the evidence on modifiable risk factors for cognitive decline and dementia: a population‐based perspective. Alzheimers Dement 2015; 11: 718–726. 10.1016/j.jalz.2015.05.016. 26045020

[46] Jia F , Liu F , Li X , Shi X , Liu Y , Cao F . Cognitive reserve, modifiable‐risk‐factor profile and incidence of dementia: results from a longitudinal study of CFAS Wales. Aging Ment Health 2021; 25: 2286–2292. 10.1080/13607863.2020.1828270. 33021096

[47] Nelson ME , Jester DJ , Petkus AJ , Andel R . Cognitive reserve, Alzheimer's neuropathology, and risk of dementia: a systematic review and meta‐analysis. Neuropsychol Rev 2021; 31: 233–250. 10.1007/s11065-021-09478-4. 33415533 PMC7790730

[48] Bassendine MF , Taylor‐Robinson SD , Fertleman M , Khan M , Neely D . Is Alzheimer's disease a liver disease of the brain? J Alzheimers Dis 2020; 75: 1–14. 10.3233/JAD-190848. 32250293 PMC7306895

[49] Levine KS , Leonard HL , Blauwendraat C *et al*. Virus exposure and neurodegenerative disease risk across national biobanks. Neuron 2023; 111: 1086–1093. 10.1016/j.neuron.2022.12.029. 36669485 PMC10079561

[50] Lin C , Chien W , Chung C *et al*. Increased risk of dementia in patients with genital warts: a nationwide cohort study in Taiwan. J Dermatol 2020; 47: 503–511. 10.1111/1346-8138.15277. 32189395

[51] Marcocci ME , Napoletani G , Protto V *et al*. Herpes simplex Virus‐1 in the brain: the dark side of a sneaky infection. Trends Microbiol 2020; 28: 808–820. 10.1016/j.tim.2020.03.003. 32386801

[52] Muzambi R , Bhaskaran K , Brayne C , Davidson JA , Smeeth L , Warren‐Gash C . Common bacterial infections and risk of dementia or cognitive decline: a systematic review. J Alzheimers Dis 2020; 76: 1609–1626. 10.3233/JAD-200303. 32651320 PMC7504996

[53] Chu C‐S , Liang C‐S , Tsai S‐J *et al*. Bacterial pneumonia and subsequent dementia risk: a nationwide cohort study. Brain Behav Immun 2022; 103: 12–18. 10.1016/j.bbi.2022.04.002. 35390468

[54] Nayeri T , Sarvi S , Sharif M , Daryani A . Toxoplasma gondii: a possible etiologic agent for Alzheimer's disease. Heliyon 2021; 7: e07151. 10.1016/j.heliyon.2021.e07151. 34141920 PMC8187970

[55] Yang H‐Y , Chien W‐C , Chung C‐H *et al*. Risk of dementia in patients with toxoplasmosis: a nationwide, population‐based cohort study in Taiwan. Parasit Vectors 2021; 14: 435. 10.1186/s13071-021-04928-7. 34454590 PMC8401101

[56] Alzheimer's Association . Myths, n.d. Available from URL: https://www.alz.org/alzheimers-dementia/what-is-alzheimers/myths.

[57] Nagel AK , Loetscher T , Smith AE , Keage HA . What do the public really know about dementia and its risk factors? Dementia 2021; 20: 2424–2440. 10.1177/1471301221997301. 33745347

[58] Mishra P , Pandey C , Singh U , Gupta A , Sahu C , Keshri A . Descriptive statistics and normality tests for statistical data. Ann Card Anaesth 2019; 22: 67–72. 10.4103/aca.ACA_157_18. 30648682 PMC6350423

[59] Mondschein CF , Monda C . The EU's general data protection regulation (GDPR) in a research context. In: Kubben P , Dumontier M , Dekker A , eds. Fundamentals of Clinical Data Science. Cham: Springer International Publishing, 2019; 55–71. 31314241

[60] Goncharova G , Karamelska T . Care of People Living with dementia in Bulgaria: between over‐responsibility to the family and distrust in public health services and policies. Comp Southeast Eur Stud 2024; 72: 58–82. 10.1515/soeu-2023-0015.

[61] Van Patten R , Tremont G . Public knowledge of late‐life cognitive decline and dementia in an international sample. Dementia 2020; 19: 1758–1776. 10.1177/1471301218805923. 30309254

[62] Al‐Awad FA , AlAbdulkader A , Shammari MA *et al*. Knowledge levels and sociodemographic influences on dementia awareness in the Eastern Province of Saudi Arabia. Electron J Gen Med 2024; 21: em567. 10.29333/ejgm/14159.

[63] Pacifico D , Fiordelli M , Fadda M *et al*. Dementia is (not) a natural part of ageing: a cross‐sectional study on dementia knowledge and misconceptions in Swiss and Italian young adults, adults, and older adults. BMC Public Health 2022; 22: 2176. 10.1186/s12889-022-14578-8. 36434540 PMC9701025

[64] Creten S , Heynderickx P . Dementia literacy and its link to public attitudes towards dementia in Flanders: a cross‐sectional survey among health professionals, family caregivers, and the general public. Open J Soc Sci 2024; 12: 84–99. 10.4236/jss.2024.122006.

[65] Eccleston CE , Courtney‐Pratt H , McInerney F , Johnstone A , Doherty K . Predictors of dementia knowledge in a rural general public sample. Aust J Rural Health 2021; 29: 530–537. 10.1111/ajr.12777. 34351673

[66] Danailova Petrova‐Geretto E , Yanakieva A , Vodenicharova A . Health literacy: a call for action for a just and egalitarian society. Sci Int J 2023; 2: 37–40. 10.35120/sciencej020137d.

[67] Sørensen K , Pelikan JM , Röthlin F *et al*. Health literacy in Europe: comparative results of the European health literacy survey (HLS‐EU). Eur J Public Health 2015; 25: 1053–1058. 10.1093/eurpub/ckv043. 25843827 PMC4668324

[68] OECD, European Observatory on Health Systems and Policies . Bulgaria: Country Health Profile 2023 Paris: State of Health in the EU, OECD Publishing, Paris, 2023. 10.1787/8d90f882-en.

[69] National Statistical Institute of Bulgaria . Poverty and Social Inclusion Indicators in 2022. Sofia: National Statistical Institute of Bulgaria, 2022. https://www.nsi.bg/sites/default/files/files/pressreleases/SILC2022_en_4UPGZS4.pdf (accessed 29 Oct 2024).

[70] World Health Organization (WHO) . Global action plan on the public health response to dementia 2017‐2025. World Health Organization, 2017. https://www.who.int/publications/i/item/9789241513487 (accessed 10 Jan 2025).

