# Supplementary material for: Gaps in dementia knowledge: a nationwide study of public awareness and misconceptions in Bulgaria
Source: Psychogeriatrics. 2025 Feb 27;25(2):e70016. doi: 10.1111/psyg.70016 (PMC11868682; doi:10.1111/psyg.70016)
Supplement: Supplementary file 1 — Table S1. Results from χ 2 tests of independence assessing the connection between vignette recognition ability and sociodemographic factors, previous experience with dementia, and number and quality of used informational sources. The results show that the only factors associated with the ability to recognise the symptoms of dementia from a vignette are having professional contact with dementia patients and the number of used informational sources. Table S2. Extended contingency table for outcomes of vignette recognition task and having professional contact with dementia patients. Results from the post hoc analysis of Pearson's χ 2 test residuals are included in the bottom section of the table. Statistical significance is indicated as follows: *P < 0.05; **P < 0.01; ***P < 0.001. Table S3. Extended contingency table for outcomes of vignette recognition task and the number of used informational sources about dementia. Results from the post hoc analysis of Pearson's χ 2 test residuals are included in the bottom section of the table. Statistical significance is indicated as follows: *P < 0.05; **P < 0.01; ***P < 0.001. Table S4. Group differences in Dementia Knowledge Assessment Scale (DKAS) scores. Groups are formulated on the basis of sociodemographic characteristics, previous experience with dementia, used informational sources about dementia, and vignette recognition. Table S5. Results from analyses of recognised dementia risk factors in terms of sociodemographic characteristics, previous experience with dementia, and used informational sources about dementia. Table S6. Results from two‐tailed binomial tests for items with emerging evidence. [file PSYG-25-0-s001.docx]

*Table S1: Results from chi-squared tests of independence assessing the connection between vignette recognition ability and sociodemographic factors, previous experience with dementia, and number and quality of used informational sources. The results show that the only factors associated with the ability to recognize the symptoms of dementia from a vignette are having professional contact with dementia patients and the number of used informational sources.*

|  | chi-squared | df | p-value |
| --- | --- | --- | --- |
|  | *Socio-demographic characteristics* | | |
| Gender | 1.1175 | 1 | 0.2905 |
| Age group | 0.17196 | 2 | 0.9176 |
| Education | 8.9879 | 4 | 0.0614 |
| Employment status | 3.0754 | 4 | 0.5453 |
| Marital status | 3.5764 | 4 | 0.4664 |
| Region of residence | 4.9987 | 5 | 0.4160 |
| Living arrangement | 0.096408 | 1 | 0.7562 |
| Location | 2.6839 | 2 | 0.2613 |
| Ethnicity | 5.6807 | 4 | 0.2243 |
|  | *Previous experience with dementia* | | |
| Experience as a caretaker | 0.18064 | 1 | 0.6708 |
| Having a sick relative | 0.0025722 | 1 | 0.9596 |
| Profession involves contact with sick individuals | 6.016 | 1 | **0.0142 **** |
|  | *Informational sources* |  |  |
| Number of informational sources  (<3, 3-5, 6-9) | 8.1051 | 2 | **0.0173*** |
| Using formal informational sources | 6.5687 | 1 | 0.8631 |

*Table S2: Extended contingency table for outcome of vignette recognition task and having professional contact with dementia patients. Results from the post hoc analysis of Pearson’s Chi Squared Test residuals are included in the bottom section of the table. Statistical significance is indicated as follows: *** p < .001; ** p <.01; * p < .05*

|  | *Professional contact (YES)* | *Professional contact (NO)* |
| --- | --- | --- |
|  | n | |
| *Vignette Failure* | 24 | 924 |
| *Vignette Success* | 45 | 903 |
|  | Residuals of Person’s Chi Squared Test | |
| *Vignette Failure* | -2.57  *(p* =.040*) | 2.57  *(p* =.040*) |
| *Vignette Success* | 2.57  *(p* =.040*) | -2.57  *(p* =.040*) |

*Table S3:* *Extended contingency table for outcome of vignette recognition task and number of used informational sources about dementia. Results from the post hoc analysis of Pearson’s Chi Squared Test residuals are included in the bottom section of the table. Statistical significance is indicated as follows: *** p < .001; ** p <.01; * p < .05*

|  | *Number of informational sources* | | |
| --- | --- | --- | --- |
|  | ***< 3*** | ***3-5*** | ***6-9*** |
|  | *n* | | |
| *Vignette Failure* | 640 | 300 | 8 |
| *Vignette Success* | 581 | 356 | 11 |
|  | *Residuals of Person’s Chi Squared Test* | | |
| *Vignette Failure* | 2.83  (*p* = .028*) | -2.70  (*p* = .041*) | -0.69  (*p* = 1.00) |
| *Vignette Success* | -2.83  (*p* = .028*) | 2.70  (*p* = .041*) | 0.69  (*p* = 1.00) |

*Table S4: Group differences in DKAS scores. Groups are formulated on the basis of sociodemographic characteristics, previous experience with dementia, used informational sources about dementia, and vignette recognition.*

| **Variables** | **ANOVA (mean DKAS scores)** | | | | | |  |
| --- | --- | --- | --- | --- | --- | --- | --- |
|  | *F value* | | | *df* | *p-value* | |  |
| Age group | 0.2639 | | | 2,1893 | 0.7808 | |  |
| Education | 14.6635 | | | 4, 1891 | < .001 *** | |  |
| Employment status | 15.8129 | | | 4, 1891 | < .001 *** | |  |
| Marital status | 10.377 | | | 4, 1891 | < .001 *** | |  |
| Region of residence | 1.7566 | | | 5, 1890 | 0.1186 | |  |
| Living arrangement | 0.3805 | | | 1, 1894 | 0.5373 | |  |
| Location | 1.0398 | | | 2,1893 | 0.3537 | |  |
| Ethnicity | 12.1708 | | | 4, 1891 | < .001 *** | |  |
| Number of informational sources  (<3, 3-5, 6-9) | 58.0654 | | | 2, 1893 | < .001 *** | |  |
|  | **Two-tailed Welch’s T-tests (mean DKAS scores)** | | | | | |  |
|  | *Levels*  *Group Means* | | | *df* | *T statistic* | *p-value* | |
| Gender | Female  9.5682 | Male  9.4485 | | 1858.05 | -0.5575 | 0.5771 | |
| Experience as a caretaker | Yes  10.9024 | | No  9.2194 | 488.99 | 6.1928 | < .001*** | |
| Having a sick relative | Yes  10.7802 | | No  8.9970 | 1179.94 | 8.2162 | < .001*** | |
| Profession involves contact with sick individuals | Yes  12.4638 | | No  9.3990 | 76.03 | 6.5644 | < .001*** | |
| Using formal informational sources | Yes  11.2777 | | No  9.0705 | 661.82 | -9.2503 | < .001*** | |
| Successful vignette recognition | Yes  10.0200 | | No  9.0010 | 1889.63 | -4.7916 | < .001*** | |

*Table S5: Results from analyses of recognized dementia risk factors in terms of sociodemographic characteristics, previous experience with dementia, and used informational sources about dementia.*

| **Variables** | **ANOVA (number of correctly identified risk factors)** | | | | | |  |
| --- | --- | --- | --- | --- | --- | --- | --- |
|  | *F value* | | | *df* | *p-value* | |  |
| Age group | 4.3388 | | | 2,1893 | 0.0131* | |  |
| Education | 15.9078 | | | 4, 1891 | < .001 *** | |  |
| Employment status | 19.6383 | | | 4, 1891 | < .001 *** | |  |
| Marital status | 10.4372 | | | 4, 1891 | < .001 *** | |  |
| Region of residence | 0.3106 | | | 5, 1890 | 0.9068 | |  |
| Living arrangement | 0.2550 | | | 1, 1894 | 0.6136 | |  |
| Location | 0.8716 | | | 2,1893 | 0.4185 | |  |
| Ethnicity | 15.4240 | | | 4, 1891 | < .001 *** | |  |
| Number of informational sources  (<3, 3-5, 6-9) | 6.5687 | | | 2, 1893 | 0.0014 ** | |  |
|  | **Two-tailed Welch’s T-tests (number of correctly identified risk factors)** | | | | | |  |
|  | *Levels*  *Group Means* | | | *df* | *T statistic* | *p-value* | |
| Gender | Female  7.5978 | Male  7.4048 | | 1889.53 | -1.0860 | 0.2776 | |
| Experience as a caretaker | Yes  7.4238 | | No  7.5217 | 522.55 | -0.4550 | 0.6493 | |
| Having a sick relative | Yes  7.5293 | | No  7.4948 | 1311.58 | 0.1967 | 0.8441 | |
| Profession involves contact with sick individuals | Yes  8.9565 | | No  7.4499 | 77.32 | 4.1322 | < .001*** | |
| Using formal informational sources | Yes  8.1560 | | No  7.3426 | 677.88 | -4.1058 | < .001*** | |

*Table S6: Results from two-tailed binomial tests for items with emerging evidence.*

*Estimated proportions for “Contributes” answers were tested against chance (.50). P values <.05 signify different from chance probabilities of answering “Contributes” for a particular item.*

*Answer categories were formulated as follows:*

*Contributes = the sum of all “contributes” and “probably contributes” answers.*

*Does not contribute = the sum of all “does not contribute” and ”probably does not contributes” answers plus all neutral answers.*

| Factor | Answer Categories | n | Estimated  Proportions | Test Proportion | p  (two-tailed) | Direction of  effect |
| --- | --- | --- | --- | --- | --- | --- |
| Virus | Contributes  Does not contribute | 1158  738 | 0.61  0.29 | .50 | < .001 | Correct |
| Bacterial infection | Contributes  Does not contribute | 1109 787 | 0.58  0.42 | .50 | < .001 | Correct |
| Parasite | Contributes  Does not contribute | 780 1116 | 0.41  0.59 | .50 | < .001 | Incorrect |
